# Postpartum care screenings by care modality among US mothers, 2020–2021

**DOI:** 10.1016/j.xagr.2025.100541

**Published:** 2025-07-03

**Authors:** Don E. Willis, Cari A. Bogulski, Clare C. Brown, Nirvana A. Manning, Lanita S. White, James P. Selig, Ji Li, Pearl A. McElfish

**Affiliations:** a Institute for Community Health Innovation, University of Arkansas for Medical Sciences Northwest, Springdale, AR (Willis and McElfish); b College of Medicine, University of Arkansas for Medical Sciences Northwest, Springdale, AR (Willis and McElfish); c College of Medicine, University of Arkansas for Medical Sciences Northwest, Fayetteville, AR (Bogulski); d Fay W. Boozman College of Public Health, University of Arkansas for Medical Sciences, Little Rock, AR (Brown); e College of Medicine, University of Arkansas for Medical Sciences, Little Rock, AR (Manning); f College of Pharmacy, University of Arkansas for Medical Sciences, Little Rock, AR (White); g Fay W. Boozman College of Public Health, University of Arkansas for Medical Sciences Northwest, Springdale, AR (Selig and Li)

**Keywords:** abuse, depression, postpartum care, screenings, smoking, virtual care

## Abstract

**BACKGROUND:**

Postpartum care is critical to preventing pregnancy-associated deaths. Virtual modes of care have potential to improve access to postpartum care; however, the impact on postpartum screenings for cigarette smoking, emotional/physical abuse, and depression is unknown.

**OBJECTIVE:**

The purpose of this study was to compare receipt of these screenings between mothers who received any virtual postpartum care and those whose care was exclusively in-person.

**STUDY DESIGN:**

Using the Pregnancy Risk Assessment Monitoring System Phase 8 data, we estimated receipt of postpartum care screenings for smoking, emotional/physical abuse, and depression for US mothers during COVID-19 (2020–2021) and assessed differences by mode of care. The analytical sample (n=21,815) included mothers from 28 Pregnancy Risk Assessment Monitoring System study sites who had postpartum care and answered the mode of care question and all 3 postpartum screening questions.

**RESULTS:**

Over half of all mothers were screened for cigarette smoking (56.45%) and emotional/physical abuse (59.28%) during their postpartum care, whereas screening for depression was much more prevalent (87.63%). A higher percentage of screenings for smoking, emotional/physical abuse, and depression was reported for mothers who received any virtual postpartum care compared with those who attended exclusively in-person care (all *P*<.001). After adjusting for covariates, the prevalence of screening for cigarette smoking, emotional/physical abuse, and depression was 14%, 18%, and 6% higher, respectively, among mothers who received any virtual (vs. exclusively in-person) postpartum care.

**CONCLUSION:**

Virtual postpartum care may improve the percentage of women receiving screenings for important maternal health risks and behaviors, such as cigarette smoking, emotional/physical abuse, and depression. Further research is needed to determine whether mothers receiving virtual postpartum care are more likely to receive screenings because of the mode of care itself.

**AJOG Global Reports at a Glance**

**Why was this study conducted?**

This study aimed to advance knowledge of how virtual modes of care may influence postpartum care screenings by comparing receipt of screenings between mothers who received any virtual postpartum care and those whose care was exclusively in-person.

**Key findings**

Mothers who received any virtual postpartum care had greater odds of being screened for smoking, emotional/physical abuse, and depression relative to those who received exclusively in-person postpartum care.

**What does this add to what is known?**

This study compared receipt of recommended postpartum screenings between mothers who received any virtual postpartum care and those who received exclusively in-person care. It offers insights into how virtual postpartum care strategies might increase receipt of critical screenings.

## Introduction

Over 80% of pregnancy-related deaths (ie, deaths from medical or obstetrical causes) are preventable, and over half occur between 7 and 365 days after delivery, making postpartum care vital to the lives of mothers and their dependents.^1^ Suicide, homicide, and substance use are the leading causes of pregnancy-associated deaths (ie, deaths during pregnancy or up to 1 year postpartum from any cause),^2^^,^^3^ highlighting the critical importance of screening postpartum patients for mental health conditions (eg, depression), behaviors (eg, cigarette smoking), and other risk factors (eg, emotional/physical abuse). Accordingly, the American College of Obstetricians and Gynecologists (ACOG) recommends that postpartum care include screenings for cigarette smoking, substance abuse, emotional/physical abuse, anxiety, and depression.^4^^,^^5^ Screening for depression during the postpartum period is also recommended by existing national quality standards, which are often tied to financial incentives.^6^ ACOG also recommends performance of glucose screening for women with gestational diabetes mellitus and a well-woman screening (ie, Papanicolaou smear and pelvic examination).^5^

Postpartum care in the United States (US) has commonly consisted of 1 visit between 4 and 6 weeks after delivery.^7^ However, recent ACOG guidance has proposed approaching postpartum care as a continuum beginning with clinician contact within 3 weeks of delivery, a comprehensive visit by 12 weeks after delivery, and a transition to ongoing well-woman care.^5^^,^^8^ Postpartum visit attendance rates vary substantially, ranging from 25.9% to 96.5%, with higher rates observed in self-reported data sources such as the Pregnancy Risk Assessment Monitoring System (PRAMS).^9^^,^^10^ Given the importance of postpartum care for maternal health, many studies have focused on disparities in access.^11^^,^^12^ Racial disparities in postpartum care have been well-documented,^11^^,^^12^ and recent research suggests that these disparities were exacerbated during the COVID-19 pandemic.^12^ Although it must be implemented thoughtfully,^13^^,^^14^ some scholars have noted the potential of virtual care to reduce or even eliminate disparities in postpartum care across social determinants of health.^14^^,^^15^ Compared with exclusively in-person care, use of virtual maternal care strategies generally results in similar or sometimes better clinical and patient-reported outcomes.^16^, ^17^, ^18^ Fewer studies have examined receipt of recommended postpartum care content by mode of care.

Recent evidence suggests that fewer than half of comprehensive postpartum visits conducted in person included recommended services, including but not limited to screenings.^19^ Evidence varies substantially regarding postpartum care *screenings specifically*. For example, although postpartum care providers report that they screen for depression almost “always” and for cigarette smoking and emotional/physical abuse between “sometimes” and “always,”^20^ evidence from PRAMS (2016–2019) suggests that just over half of the mothers are screened for cigarette smoking and emotional/physical abuse, whereas over 80% are screened for depression.^6^ Other studies report screening rates for depression during comprehensive postpartum visits as low as 8.7%.^19^ Screenings for depression, cigarette smoking, and emotional/physical abuse also vary by patient identities^21^ and geographic location.^22^ For example, maternal age, race/ethnicity, insurance status, and rurality are frequently the strongest predictors of whether patients received the recommended postpartum screenings.^6^^,^^21^, ^22^, ^23^ Previous studies often found that privately insured, White, urban residents are less likely to receive screenings for depression, cigarette smoking, and emotional/physical abuse than Medicaid-insured, racially minoritized, rural residents.^6^^,^^21^, ^22^, ^23^ A single study found receipt of recommended services to be similar across insurance types.^19^

Although scholars have argued that virtual care has potential to prevent pregnancy-associated deaths and reduce disparities in postpartum care,^14^^,^^15^^,^^24^^,^^25^ *no studies to our knowledge have compared receipt of recommended screening between individuals receiving exclusively in-person care and those receiving any virtual postpartum care.* A single-site study found both greater use of virtual postpartum care and a decline in depression screening during “peak-COVID” (ie, February to April 2020) and noted that the nurses who typically conducted intake assessment no longer performed the screening during virtual visits.^26^ This previous study provides important lessons regarding the necessary integration of screenings into virtual postpartum care, but it did not directly compare receipt of depression screenings by mode of postpartum care visit. Other parallel evidence found lower odds of Papanicolaou smear screenings among patients who exclusively used virtual postpartum care.^27^ However, Papanicolaou tests require physical examination/in-person interaction, and the study did not assess receipt of the recommended screenings for cigarette smoking, emotional/physical abuse, and depression, which can occur virtually. The purpose of this study is to fill this gap in the literature by assessing differences in postpartum care screenings by mode of care for mothers living in the US who gave birth during COVID-19 (2020–2021).

## Methods

We used the PRAMS Phase 8 survey and birth certificate data from the Automated Research File and the Maternal COVID-19 Experiences supplemental questionnaire. PRAMS is a population-based surveillance system administered by health departments and the Centers for Disease Control and Prevention. Although the PRAMS questionnaire does not collect information on gender identity,^28^ we use the term “mother” to align with the birth certificate files that provide the sampling frame for PRAMS data collection.^29^ Mothers are sampled between 8 and 16 weeks after giving birth. Forty-six states plus Washington, DC, Puerto Rico, and New York City act as PRAMS study sites, and the surveillance system has been estimated to cover 83% of all US births.^30^ PRAMS surveys are administered primarily via mail, with follow-up telephone calls made to nonrespondents. Surveys are completed in 20 to 30 minutes.^30^ Most PRAMS survey items have nonresponse rates ranging between 1% and 2%.^30^ The University of Arkansas for Medical Sciences Institutional Review Board determined this study was not human subject research given the use of data provided by a third party without information allowing reidentification (#297544).

The Maternal COVID-19 Experiences supplemental questionnaire was administered during COVID-19 and contains our primary independent variable of interest (mode of postpartum care delivery). Our analytical sample included PRAMS study sites that used this supplemental questionnaire in 2020 or 2021 and, in turn, asked mothers about mode of postpartum care. The Figure displays the geographic coverage of our analytical sample, including 28 sites across the US, including 25 US states (AK, AR, AZ, DE, GA, IA, IL, LA, MA, MD, MI, MO, ND, NE, NJ, NY, OR, PA, SD, TN, UT, VA, VT, WV, WY); Puerto Rico; Washington, DC; and New York City. The average weighted response rate was 55.7% for all sites/years included in the analytical sample. Mothers surveyed at those sites who answered the question about mode of postpartum care (n=28,216) and indicated receipt of some type of postpartum care (n=25,804) were eligible for inclusion in the analytical sample. Most mothers who answered the mode of care question reported receiving postpartum care (92%). The final analytical sample included those who had postpartum care and answered both the mode of care question and all 3 screening questions (n=21,815).

**Figure:**
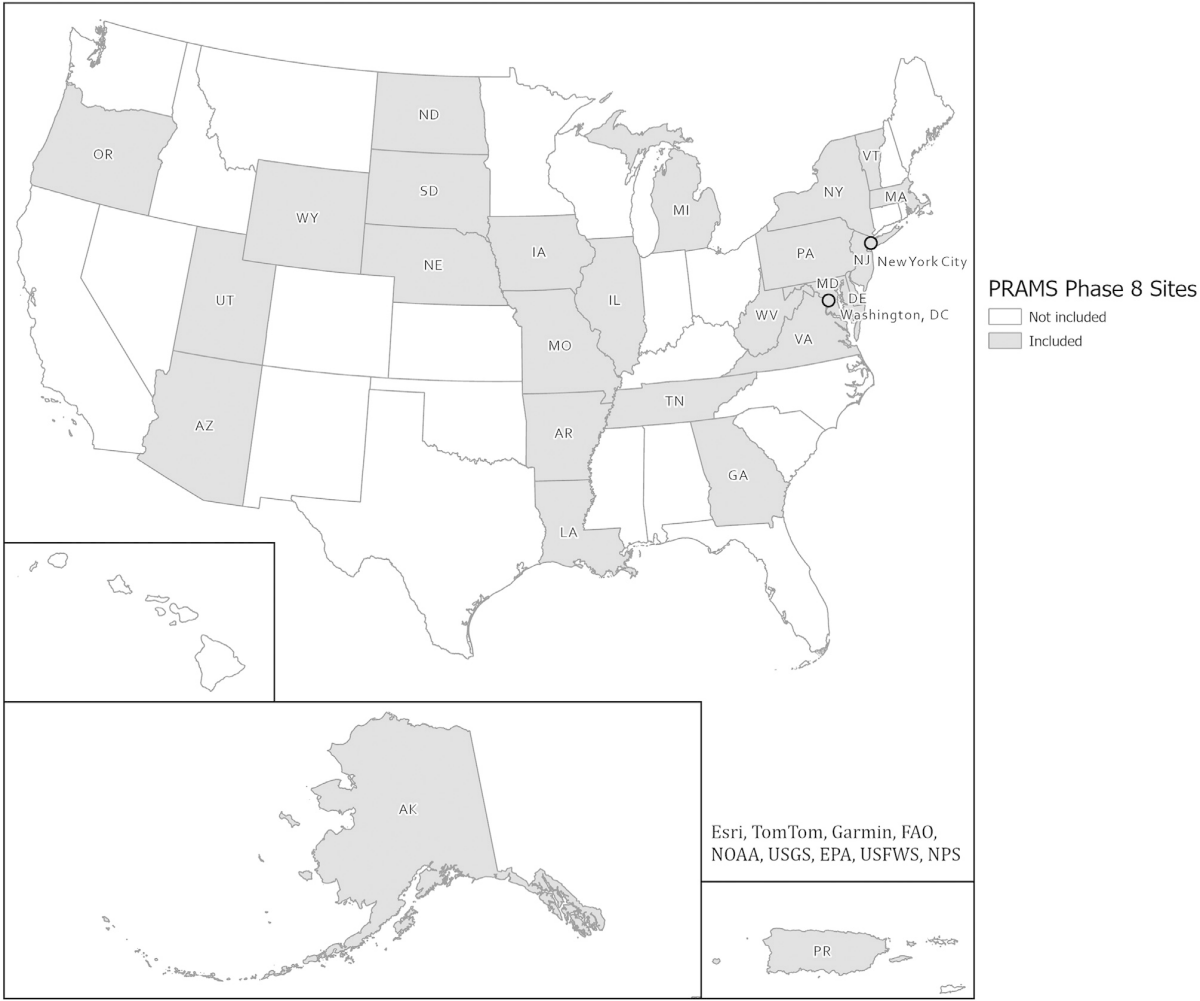
Geographic coverage of analytical sample (n=21,815) *PRAMS*, Pregnancy Risk Assessment Monitoring System.

Willis. Postpartum care screenings by care modality. Am J Obstet Gynecol Glob Rep 2025.

### Measures

#### Outcome variables: postpartum care screenings

Respondents were asked to indicate “yes” or “no” to 3 postpartum screening questions following the prompt: “During your postpartum checkup, did a doctor, nurse, or other health care worker do any of the following things?” These included: “ask me if I was smoking cigarettes,” “ask me if someone was hurting me emotionally or physically,” and “ask me if I was feeling down or depressed.” Some studies have referred to the second item as screening for intimate partner violence.^21^ We refer to it as emotional/physical abuse screening because the survey item does not specifically refer to harm from an intimate partner.

#### Primary independent variable: postpartum care modality

*Virtual postpartum care* receipt was assessed by asking, “During the COVID-19 pandemic, which types of postpartum appointments did you attend for yourself?” Response options included, “in-person appointments only,” “virtual appointments (video or telephone) only,” “both, in-person and virtual appointments,” and “I did not have any postpartum appointments for myself.” From these responses, we generated a dichotomous variable (any virtual=1; exclusively in-person=0) as prior studies have done to make our results more comparable to extant literature.^31^^,^^32^

#### Covariates

##### Sociodemographic characteristics

*Maternal age* was calculated in years from the mother’s date of birth and categorized into <25, 25 to 34, and ≥35 years. Combining responses from race and ethnicity survey questions, *race/ethnicity* was categorized into 5 mutually exclusive racial/ethnic groups: non-Hispanic White (White), non-Hispanic Asian (Asian), non-Hispanic Black (Black), non-Hispanic other race/multiracial (other/multiracial), and Hispanic. Birth certificate files provided information on *marital status* and *educational attainment.* Marital status was categorized as married or not married. Education was categorized into 4 categories: less than high school, high school graduate, some college/associate degree, and bachelor’s degree or higher. *Prepregnancy insurance status* was determined by asking respondents about the type of insurance they had in the month before pregnancy and was categorized as insured or not insured. The measure of *rural residence* was constructed on the basis of the National Center for Health Statistics Urban-Rural Classification Scheme for Counties.^33^

##### Pregnancy characteristics

Analytical variables of *plurality* (singleton or multiple), *previous live births* (0, 1, 2, 3+), and *year of infant birth* (2020 or 2021) were generated from birth certificate information by PRAMS in the Automated Research File.

### Statistical analysis

We used SAS 9.4 (SAS Institute, Cary, NC)^34^ for descriptive statistics weighted for the complex survey design and applied full information maximum likelihood (FIML) estimation^35^ with Mplus Version 8 (Muthén & Muthén, Los Angeles, CA)^36^ to minimize the limitations of missing data. Statistical significance was determined at an α level of .05 for all analyses. We tested for differences in receipt of postpartum care screenings by sociodemographic factors, pregnancy characteristics, and mode of postpartum care using Rao-Scott chi-square tests.^37^^,^^38^ FIML was used to estimate a multivariable logistic regression for each of the 3 screening outcomes to minimize the limitations of missing data while estimating adjusted risk ratios.

## Results

Table 1 displays weighted descriptive statistics of the analytical sample (n=21,815), including sociodemographic characteristics, pregnancy characteristics, and postpartum mode of care for US mothers who received postpartum care and gave birth in 2020 or 2021. Over half of the mothers were aged between 25 and 34 years. Most mothers were non-Hispanic White, married, insured before pregnancy, and urban residents. Fewer than half had obtained a bachelor’s degree, but this was the most common level of education. Year of infant birth was nearly evenly split between 2020 and 2021. Almost all pregnancies were singleton. Two-fifths of the mothers experienced their first live birth, and a third experienced their second live birth. Over four-fifths of the mothers received postpartum care exclusively in person; however, over a tenth received some postpartum care virtually.

**Table 1:** Mothers who received postpartum care, Pregnancy Risk Assessment Monitoring System 2020–2021

Variables	Weighted descriptives		
	Total	Any virtual PPC	
Sociodemographic	Col. %	Row %	Rao-Scott χ^2^
Maternal age (n=20,309), y			0.003
17–24	18.67	14.39	
25–34	59.24	15.17	
≥35	22.09	18.13	
Race/ethnicity (n=20,129)			*P*<.001
NH Asian	6.16	20.97	
NH Black	12.53	15.06	
NH White	57.67	13.83	
NH other/multiracial	3.09	17.72	
Hispanic	20.55	18.50	
Marital status (21,806)			0.789
Not married	34.09	15.69	
Married	65.91	15.90	
Educational attainment (n=21,665)			*P*<.001
Less than high school	8.42	17.99	
High school graduate	22.99	15.79	
Some college/Associate degree	24.14	12.72	
Bachelor’s degree or higher	44.45	17.05	
Prepregnancy insured status (n=21,800)			0.048
Insured	89.49	16.12	
No insurance	10.51	13.38	
Rural residence (n=19,142)			*P*<.001
Urban	88.08	17.32	
Rural	11.92	10.63	
Pregnancy characteristics			
Year of infant birth (n=21,815)			0.002
2020	50.62	17.02	
2021	49.38	14.63	
Plurality (n=20,728)			0.160
Singleton	98.50	15.65	
Multiples	1.50	19.63	
Previous live births (n=21,780)			0.007
0	41.81	17.15	
1	33.07	15.09	
2	15.26	16.00	
≥3	9.96	12.78	
Postpartum care			
PPC mode (n=21,815)			
Exclusively in-person	84.16		
Any virtual	15.84		

Percentages may not sum to 100 because of rounding.

*NH*, non-Hispanic; *PPC*, postpartum care.

Table 2 includes percentages of mothers who received screenings overall and by postpartum care modality, along with Rao-Scott chi-square tests for statistical significance of differences in screening across modalities. Just over half of the mothers were screened for cigarette smoking and emotional/physical abuse during their postpartum care, whereas screening for depression was much more prevalent. The percentage of postpartum care screenings differed significantly across modes of care, with significantly higher proportions among mothers who received any virtual postpartum care relative to those with exclusively in-person care for screening for cigarette smoking, emotional/physical abuse, and depression.

**Table 2:** Weighted percentages of mothers who received postpartum care screenings overall and by modality, Pregnancy Risk Assessment Monitoring System 2020–2021

Screening	Overall sample	Any virtual	Exclusively in-person	Rao-Scott χ^2^	
	% (LB–UB)	% (LB–UB)	% (LB–UB)	F	*P* value
Cigarette smoking (n=21,663)	56.45 (55.39–57.51)	62.82 (60.39–65.25)	55.24 (54.07–56.41)	29.14	<.001
Emotional/physical abuse (n=21,735)	59.28 (58.24–60.33)	68.71 (66.38–71.04)	57.51 (56.34–58.67)	65.11	<.001
Depression (n=21,778)	87.63 (86.93–88.32)	92.33 (91.02–93.63)	86.75 (85.96–87.53)	38.01	<.001

*LB*, lower 95% confidence bound; *UB*, upper 95% confidence bound.

Table 3 displays results for 3 separate FIML multivariable logistic regressions—1 for each postpartum care screening adjusted for sociodemographic variables and pregnancy characteristics. After adjusting for covariates, the prevalence of screening for cigarette smoking, emotional/physical abuse, and depression was 14%, 18%, and 6% higher, respectively, among mothers who received any virtual postpartum care relative to those who received exclusively in-person postpartum care.

**Table 3:** Full information maximum likelihood multivariable logistic regressions for postpartum care screenings by modality, Pregnancy Risk Assessment Monitoring System 2020–2021

Models	RR
Model 1: Cigarette smoking screening	
Any virtual	1.14
Exclusively in-person	—
Model 2: Emotional/physical abuse screening	
Any virtual	1.18
Exclusively in-person	—
Model 3: Depression screening	
Any virtual	1.06
Exclusively in-person	—

Risk ratios are adjusted for maternal age, race/ethnicity, marital status, educational attainment, prepregnancy insurance status, rural residence, year of infant birth, plurality, and previous live births.

*RR*, marginal approximation risk ratio.

## Discussion

### Principal findings

Among a multistate sample of mothers who gave birth in 2020 or 2021 and received some type of postpartum care, our results demonstrate that receiving any virtual postpartum care was associated with a higher prevalence of screening for cigarette smoking, emotional/physical abuse, and depression relative to receiving postpartum care exclusively in person.

### Results in the context of what is known

Our principal findings suggest that virtual care has potential to improve receipt of recommended postpartum care services, particularly for screenings that can be performed virtually and are related to sensitive or stigmatized subjects (eg, cigarette smoking, emotional/physical abuse, depression). Although we found no prior studies with results directly comparable to our own, the 2 most similar studies reported results suggesting that virtual care might create more challenges to the receipt of postpartum care services. One study examining postpartum screenings conducted early in the COVID-19 public health emergency (between March and November 2020) called for improved integration of screenings into virtual care because the nurses did not conduct screenings during virtual care visits, which the authors suspect was the cause of decreased screenings for depression during a period when virtual visits increased.^26^ Our findings may differ from this study because it was conducted at a single site that explicitly did not integrate screenings into its virtual care, whereas our analysis includes an analytical sample of mothers who gave birth across multiple US states and years. Another study of mothers who gave birth between July 2021 and June 2023 found *exclusive use* of virtual postpartum care to be associated with lower odds of Papanicolaou smear screenings and long-acting contraceptive use^27^; however, these are 2 services that cannot be conducted with solely virtual care. Findings from our analysis likely differ because we focused on screenings that could be conducted either in person or virtually. Although neither study includes an analysis comparable to ours, both provide some evidence that virtual postpartum care can result in reduced receipt of some postpartum care components and confirm that certain services must be conducted in person.

### Clinical implications

Although rates of postpartum care receipt vary widely depending on the data source, the literature is clear that many mothers do not receive this critically important care.^9^^,^^10^ Virtual postpartum care may offer an option for accessing care that circumvents barriers to in-person care (eg, transportation and childcare), and our findings suggest that it may also influence the content of care. Postpartum care providers have competing demands on the limited time available during postpartum visits, which can lead to inconsistency even for highly prioritized and recommended components of postpartum care.^20^ For example, although screening for intimate partner violence has been shown to be highly prioritized among postpartum care providers, it was underperformed relative to other highly prioritized components of care.^20^ Researchers studying trade-offs in postpartum care delivery have recommended implementing multiple touch points to address certain components of care immediately and in person, while dedicating a separate process to ensuring that other highly prioritized services are provided.^20^ These researchers also revealed that nearly a quarter of providers support virtual care as a complementary approach to providing more comprehensive care.^20^ Our findings provide further support for potentially using virtual care to ensure that recommended postpartum care screenings are consistently provided and suggest that this may already be occurring for mothers who receive virtual postpartum care.

### Research implications

Current literature remains limited and warrants further investigation given the importance of postpartum care screenings.^39^ Future research should build on our work to determine whether postpartum care screenings are more likely to occur during virtual visits relative to in-person visits. In addition, to better understand the mechanisms through which virtual care might improve screenings, future research should investigate the possibility of differences in postpartum care content between various types of virtual care (eg, video vs audio-only virtual care). Virtual care could allow patients more control over when or if they are seen by a provider, which might reduce assumptions based on appearance, or influence the overall comfort of both providers and patients in discussing sensitive or stigmatized subjects such as smoking, abuse, or depression. Virtual care may also allow providers more efficient use of their time, enabling them to address more priorities that might compete for time during an in-person visit. It is also possible that some types of virtual care may have automated reminders to address these screenings. In addition, virtual care may simply improve access to care, allowing providers more opportunities to conduct screenings.

### Limitations

The survey data we examined do not include multiple time points for individuals and are cross-sectional. For this reason, we could not draw conclusions regarding causality. Self-reports of screenings can be subject to recall bias, which could be worsened by any unrelated and urgent medical concerns that patients may have experienced during their postpartum period. However, postpartum screenings occur closer to the time of survey than prepregnancy and prenatal screenings, minimizing the potential for recall bias. Given the limitations of the survey data, we were unable to determine if the screenings occurred during virtual or in-person visits and were able to assess only associations between virtual visit attendance and screening receipt. The timing and number of postpartum visits were unknown and could not be accounted for as indicators of adequate postpartum care. This limited our analysis because improved timing or increasing frequency of postpartum care is one plausible explanatory pathway for the observed positive association between virtual mode of care and postpartum screenings. Survey methods are subject to some degree of nonresponse bias. However, we applied survey weights that adjust for sampling design, nonresponse, and noncoverage, and PRAMS has consistently achieved high response rates in nearly all participating study sites.^30^^,^^40^ The generalizability of this study is also limited given that the sampling methods do not include all pregnancies (only live births), and not all states administered the supplemental survey that asked respondents about their mode of postpartum care. Moreover, there were some differences between our eligible and analytical sample (ie, the eligible sample without responses missing for any of the 3 outcome variables). Although the magnitude of these differences was small (<10%), this may limit the generalizability of the findings, particularly for groups underrepresented in the analytical sample, such as those with no high school degree (8% vs 14%), who identified as Black (14% vs 16%), or who lived in an urban county (82% vs 88%). There may also be important factors unaccounted for in our analysis. For example, providers who were early adopters of virtual postpartum care may have been those who already provided high-quality postpartum care, including higher screening rates.

### Conclusions

Virtual postpartum care may improve the proportion of women receiving ACOG-recommended screenings for important maternal health risks and behaviors, such as cigarette smoking, emotional/physical abuse, and depression. However, further research is needed to determine whether mothers receiving virtual postpartum care are more likely to receive screenings because of the mode of care itself (ie, at a virtual visit) or as a result of other antecedent or confounding factors unaccounted for in this study. Given that some care cannot be delivered virtually and we still do not fully understand the influence of virtual care on care content, virtual postpartum care remains best suited as a complementary approach to providing more comprehensive care in combination with in-person visits.

## CRediT authorship contribution statement

**Don E. Willis:** Writing – original draft, Formal analysis, Data curation, Conceptualization. **Cari A. Bogulski:** Writing – review & editing, Validation, Conceptualization. **Clare C. Brown:** Writing – review & editing, Conceptualization. **Nirvana A. Manning:** Writing – review & editing, Conceptualization. **Lanita S. White:** Writing – review & editing, Conceptualization. **James P. Selig:** Writing – review & editing, Supervision, Formal analysis. **Ji Li:** Writing – review & editing, Formal analysis, Data curation. **Pearl A. McElfish:** Writing – review & editing, Supervision, Resources, Funding acquisition, Conceptualization.
